# Use of the New ICD-10 Vision Codes Among Medicare Beneficiaries With Stroke

**DOI:** 10.1093/geroni/igaa057.721

**Published:** 2020-12-16

**Authors:** Kimberly Hreha, Kenneth Ottenbacher, Joshua Ehrlich, Heather Whitson

**Affiliations:** 1 The University of Texas Medical Branch at Galveston, Galveston, Texas, United States; 2 University of Michigan, Ann Arbor, Michigan, United States; 3 Duke University, Durham, North Carolina, United States

## Abstract

Older adults can experience vision impairment following stroke in combination with pre-existing ophthalmologic disease. The new ICD-10 coding system for identifying vision related health conditions provides a much higher level of detail for coding these complex scenarios than the previous ICD-9 system. While this new coding system has advantages for clinical care and billing, the degree to which providers are utilizing the expanded code structure is unknown. The study objective was to describe the use of ICD-10 vision codes in a large cohort of stroke survivors. We used a retrospective cohort design to study national 100% Medicare claims files from 2015 through 2017. Data were analyzed using all available ICD-10 vision codes for beneficiaries who had an acute care stay because of a stroke and who also had an ICD-10 visual code recorded at least once in their claims chart. The cohort (n= 269,314) was mostly female (57.1%) with ischemic stroke (87.8%). Approximately 15% were coded as having one or more vision impairments. Unspecified glaucoma was the most frequently used code among men (2.83%), beneficiaries over 85+ (4.80%) and non-Hispanic blacks (4.12%). But multiple vision codes were used in few patients, overall (0.6%). Less than 3% of those in the oldest group (85+ years) had two vision codes noted in their claims. Despite more available codes, the coding used to describe the vision impairments in this population of stroke survivors was not specific or diverse. Hospital providers should pay attention to specificity in order to improve coding practices.

